# Aged garlic extract protects against oxidative stress and renal changes in cisplatin-treated adult male rats

**DOI:** 10.1186/s12935-014-0092-x

**Published:** 2014-09-28

**Authors:** Ashraf Y Nasr, Hamid AM Saleh

**Affiliations:** Anatomy Department, Faculty of Medicine, King Abdulaziz University, P.O. Box 80205, Jeddah, 21589 Kingdom of Saudi Arabia; Anatomy Department, Faculty of Medicine, Zagazig University, Zagazig, Egypt

**Keywords:** Cisplatin, Aged garlic extract, Nephrotoxicity, Biochemical, Ultrastructure, Rats

## Abstract

**Background:**

Cisplatin (CP) is one of the effective anticancer drugs, but it causes many side effects. Aged garlic extract (AGE) is a natural herbal product used in management of many diseases.

**Objective:**

This study aimed to investigate effect of AGE on CP-induced nephrotoxicity in rats.

**Material and methods:**

Four equal groups of adult male rats: control, AGE -treated (250 mg/kg, once oral dose/ 21days), CP-treated (7.5 mg/kg once i.p. on day 16th.), combined AGE and CP-treated were used. Body and kidneys weights of each rat were calculated. Serum levels of kidney biomarkers were assessed. Malondialdehyde (MDA) and reduce glutathione (GSH) levels, superoxide dismutase (SOD) and catalase (CAT) activities of renal tissues were measured as well. Renal samples from each rat were prepared for light and electron microscopic examinations.

**Results:**

Hemorrhage, glomerular atrophy, inflammatory cell infiltration, tubular necrosis and degeneration were observed in CP-treated rats. Also, a significant (P<0.001) reduction in SOD & CAT activities, GSH levels accompanied with a significant increase in serum levels of kidney biomarkers and MDA were determined in CP-treated rats compared to control group. However, most of CP-induced histomorphological, ultrastructural and biochemical changes were improved in animals pretreated with AGE.

**Conclusion:**

Such renoprotective effect of AGE may be attributed to its antioxidant activity.

## Introduction

Cisplatin (CP) is an inorganic, divalent, water soluble, platinum-containing compound. CP is a highly efficient anti-neoplastic drug commonly used as a first-line therapy for treatment of various solid tumors such as: stomach cancer, ovarian cancer, lung cancer, bladder cancer and germ cell tumors [1]. The anticancer effect of CP is mediated by apoptosis and DNA-crosslinks with subsequent cytotoxic lesions in malignant cells [2]. However, its clinical use is associated with dose and duration-dependent nephrotoxic side effect [3]. The exact mechanism of CP-induced nephrotoxicity is not well understood. Thus, several hypotheses have been supposed to explain such effect. Generation of reactive oxygen species (ROS), impaired glutathione metabolism, alterations in the mitochondrial antioxidant enzymes such as superoxide dismutase (SOD), catalase (CAT), reduce glutathione (GSH) and increase lipid peroxidation are the most plausible mechanisms of CP-induced toxicity [4-7]. CAT and SOD are enzymes of the intrinsic antioxidant defense system, which are responsible for the dissemination of superoxide free radicals [4]. In addition, stress on endoplasmic reticulum and activation of tumor necrosis factor-α (TNF-α)-mediated apoptotic pathway have been reported as pathophysiological mechanisms in CP-induced nephrotoxicity [2].

Medicinal plants and natural herbal products have potential antioxidant activity and are therefore often administered along with chemotherapeutic agents to provide better protection against their toxic side effects [4,8,9]. Therefore, to increase the clinical usefulness of CP, various free radical scavengers have been used to ameliorate CP-induced nephrotoxicity [3,8]. Recently, much attention has been given to possible role of natural dietary antioxidants in protecting the kidney against CP-induced nephrotoxicity [8-11].

Garlic is a commonly worldwide used food and its medical properties have been well recognized for centuries [12]. Garlic and its compounds which have been reported to have diverse biological activities such as anticarcinogenic, anti-artherosclerotic, antidiabetic, renoprotective, antioxidant and immune modulation, antibacterial, antihypertensive and various other biological actions [5,12-14].

Aged garlic extract (AGE) is an odorless product resulting from prolonged extraction of fresh garlic at room temperature. AGE is sold in both dry form and as a liquid containing 10% ethanol. The process of aging gently modifies harsh and irritating compounds from the raw garlic and naturally generates unique and beneficial compounds through both enzymatic and natural chemical reactions. AGE contains various antioxidant organosulfur compounds, mainly S-Allylcysteine and allicin. These compounds play an important role as antioxidants [13]. These organosulfur compounds exert their antioxidant actions by scavenging ROS, enhancing cellular antioxidant enzymes and increasing glutathione in the cells [12,14].

AGE has been previously used to ameliorate the toxic effect of different therapeutic and toxic agents include CP [15], doxorubicin [16], acrylamide [17] and cadmium [18]. The present study has been designed to investigate the effect of AGE on the oxidative stress, biochemical and histomorphological changes of kidney in adult male Wistar albino rats treated with CP.

## Materials and methods

### Animals, chemicals and experimental design

Twenty-four adult male Wister albino rats (12–14 weeks of age) were obtained from the animal house, Faculty of Medicine, Zagazig University, Zagazig, Egypt. The rats were kept under appropriate conditions of temperature (25 ± 2°C), humidity (60–70%), light (12h dark/light cycles), free access of a commercial balanced diet and tap water ad libitum.

CP was obtained in the form of commercial Egyptian Unistin Vial (Egyptian International medical Company (EIMC) United Pharmaceuticals, Cairo, Egypt).

AGE (kyolic) was obtained from Wakunaga of America (Mission Viejo, CA). It was prepared by soaking sliced raw garlic (Allium sativum) in 15- 20% aqueous ethanol for at least 10 months at room temperature. The extract was then filtered and concentrated under reduced pressure at low temperature. The content of water-soluble compounds was relatively high while that of oil-soluble compounds was low. AGE used in this study contained 28.6% extracted solids (286 mg/ml), and S-allyl cysteine, the most abundant water-soluble compound in AGE, was present at 1.47 mg/ml.

After one week of acclimatization, the rats were randomly divided into four equal groups in separate plastic cages, six rats each. Group 1 (control) received single intraperitoneal (i.p.) dose of normal saline (0.5 ml) on day 16 and oral distilled water for 21 days; group II (AGE-treated) received single oral dose of AGE, 250 mg/kg body weight for 21 days. Groups III (CP-treated) received single i.p. dose of CP (7.5 mg/kg body weight) on day 16^th^ for inducing nephrotoxicity in rats based on pilot studies, after successive administration of oral distilled water (0.5 ml) for 21 days; group IV (combined AGE+CP-treated) received single dose of AGE (250 mg/kg body weight orally for 21 days) and single i.p. dose of CP (7.5mg/kg body weight) on day 16. The rats were weighed once every three days. LD50 of CP in rats is 12 mg/kg body weight [19]. The dose of CP was selected on the basis of its effectiveness in inducing nephrotoxicity [20].

### Samples collection

On day 22^th^, (6 days after CP injection), the rats were anesthetized by ether inhalation and a midline thoraco-abdominal incision was done to explore their viscera.

**Blood samples** were collected through a direct intracardiac puncture from each rat. The samples were poured in sterile labeled heparinized test tubes and the sere were separated and stored in a freezer at a −20°C for subsequent assessment of kidney functions.

**Tissue samples:** kidneys of each animal were excised, cleaned from their surrounding fat and connective tissue, washed with normal saline, blotted with filter paper and weighed.

**Kidney weight/body weight ratio** was calculated according to the following formula, organ weight ratio (%) = organ weight X 100/body weight [8,21].

### Assessment of kidney biomarkers

Serum creatinine, uric acid, urea and blood urea nitrogen (BUN) were measured using standard laboratory techniques and employing the commercially available diagnostic kits by an auto analyzer (BT 300, Japan) [22].

### Determination of lipid peroxidation and antioxidant enzymes

Portions from each rat’s kidneys were minced and homogenized in ice-cold phosphate buffer saline (0.05 M, pH 7.4). A part of the homogenate was mixed with an equal volume of 10% trichloroacetic acid (TCA) and centrifuged at 3000 rpm for 15 min at 4°C. Its supernatant was used to determine the content of reduced glutathione (GSH) and malondialdehyde (MDA) (indicator of lipid peroxidation) enzymes. The remaining part of the homogenate was centrifuged at 17000 × g for 60 min at 4°C and the supernatant was used for estimation the activity of superoxide dismutase (SOD) and catalase (CAT) enzymes.

Lipid peroxidation was determined by estimating the level of thiobarbituric acid reactive substances (TBARS) that was used to measure the extent of MDA formed as a result of membrane lipid peroxidation. The assay was based on the formation of a pink coloured complex in a reaction between MDA and TBARS, according to the method of Ohkawa et al., [23]; using commercial kits (Biodiagnostic, Cairo, Egypt). The colorimetric absorbance was determined at 532 nm. Specific activity was presented as nmol/mg protein. Antioxidant markers GSH, SOD, and CAT were measured by a colorimetric method using commercial kits (Biodiagnostic, Cairo, Egypt) according to the manufacturer procedures. GSH contents in kidney tissue were determined by the method of Carlberg and Mannervik [24]. Total SOD activity was determined by assaying the autoxidation and illumination of pyrogallol at 440 nm. SOD activity in kidney tissues was determined based on the ability of the enzyme to inhibit nitroblue tetrazolium (NBT) reduction by superoxide. Data were expressed as U/mg hemoglobin [25]. CAT enzyme activity was measured according to the method described by Aebi [26]. The enzymatic activity was expressed in units (1 U decomposes 1 mmol of H2O2 per minute at pH 7.0.

### Tissue processing

A. **Light Microscopy:** Specimen from each kidney was fixed in 10% neutral-buffered formalin solution for 48 hours, dehydrated in ascending grades of ethyl alcohol, cleared in xylol and embedded in paraffin blocks. Serial sections (3-5 μm) were cut using microtome (Leica RM 2125, Leica Biosystems Nussloch GmbH, Germany). The kidney sections were washed in a water bath and left in the oven for dewaxing. Thereafter, the sections were stained with hematoxylin and eosin for general histological features determination, Periodic acid Schiff (PAS) stain to demonstrate mucopolysaccharides as PAS positive [27]. The stained tissue-slides were mounted with DPX (Di-N-Butyl Phthalate Xylene) and covered with cover slips. All slides were examined by a light microscope (Olympus BH-2, Olympus, Tokyo, Japan).
B. **Electron microscopy:** Specimens of 1 mm^3^-size from the kidneys of each rat were immediately immersed in 2.5% glutaraldehyde buffered with 0.1 M phosphate buffer for 24–48 hours. Then, the specimens were washed in phosphate buffer (pH 7.2–7.4) 3–4 times for 20 min every time and post-fixed in a buffered solution of 1% osmium tetroxide for 2 h, after that washed in the same buffer 4 times for 20 min each. After fixation, the specimens were dehydrated in ascending grades of ethyl alcohol. Then, they were cleared in propylene oxide, embedded in a mixture of 1:1 of Epon resin - Araldite for 1 h. Polymerization was performed in the oven at 65°C for 24 hrs [28]. Semi-thin sections (One μm-thick) were cut with a glass knife on LKB-2000S ultramicrotome, mounted on glass slides and stained with buffered toluidine blue. The appropriate areas were selected with the light microscope. The resin blocks were trimmed to get rid of the undesired tissue. Ultrathin sections (60–90 nm thick) were cut with a glass knife on a LKB ultramicrotome, and then mounted on copper grids, double stained with uranyl acetate and lead citrate. The grids were examined and photographed using a transmission electron microscope (JEOL TEM- 1200 EX, Tokyo, Japan) operated at 60–70 kV, Faculty of Science, Ain Shams University.

### Statistical analyses

Results were expressed as mean ± SEM. Comparison of means was done by the student’s *t*-test (One way ANOVA) and the Mann–Whitney *U* test. Values of *P* < 0.05 were considered statistically significant. Statistical evaluation was conducted with SPSS version 16.0 (SPSS Inc., Chicago, IL, USA).

The study was performed after the approval of the Medical Ethical Committee of the Faculty of Medicine, Zagazig University, Zagazig City, Egypt and followed the recommendations of the National Institutes of Health Guide for Care and Use of Laboratory Animals.

## Results

No deaths were recorded in the rats of the different groups throughout the period of the study. However, a reduction in the activity and appetite with diarrhea, loss luster and hair fall were observed in the CP-treated rats.

### Body weight

**Figure 1:**
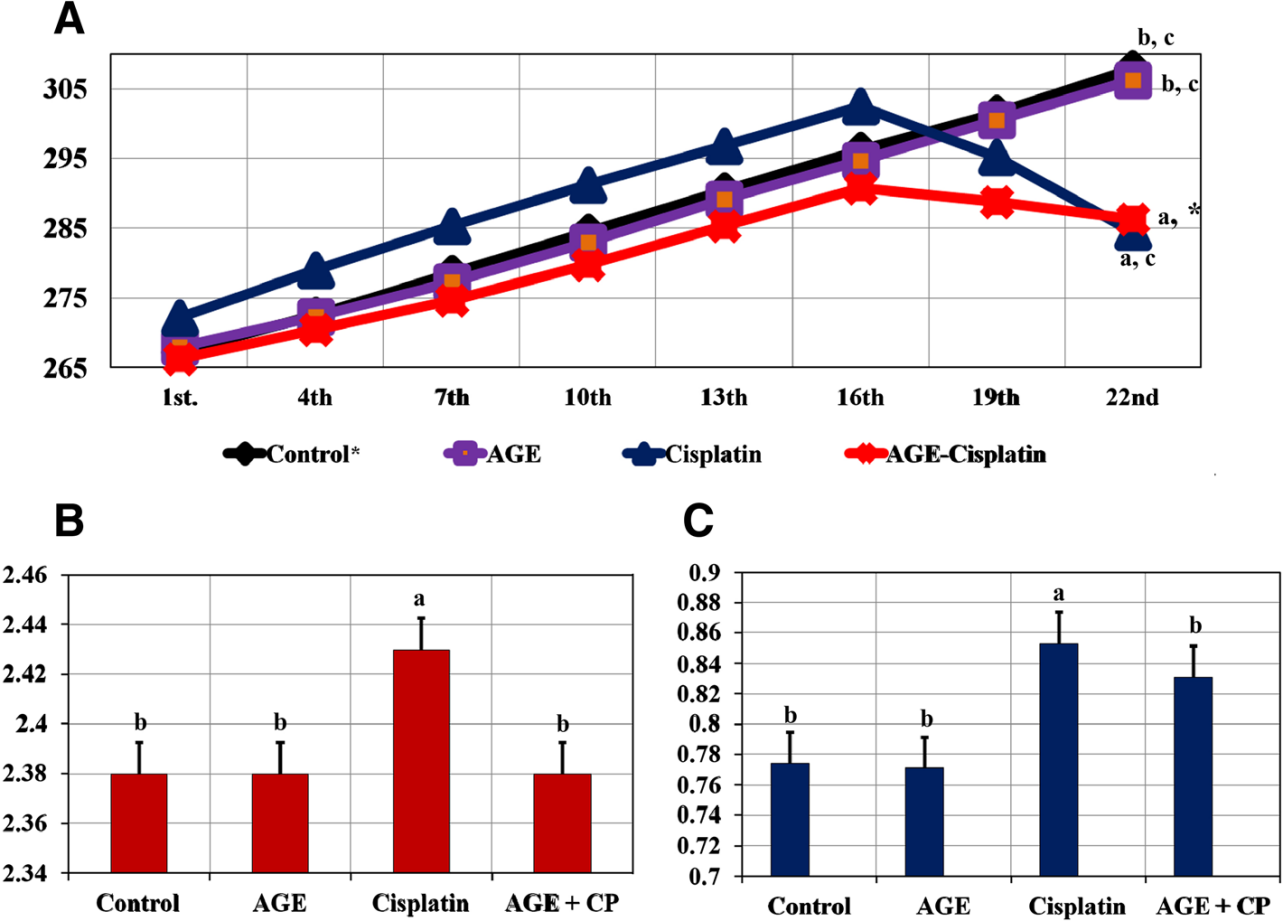
**Effect of cisplatin (CP) and / or aged garlic extract (AGE) treatment on body and kidney weights of rats.** Data are expressed as Mean ± SEM (n = 6). **A**: Significant difference of final body weight (BW) of the group Vs. those of control group at P. value < 0. 01. **B**: Significant difference between final BW of the group Vs. Final BW of cisplatin-treated group at P. value < 0.005. **C**: Significant difference between initial and final BW of same group at p. value < 0.001. *: No significant difference between initial & final BW (P = 0.197). BW: body weight; AGE: aged garlic extract; CP: cisplatin.

The effects of CP treatment with or without AGE on the body and kidney weights of rats were reported (Figure 1). The final body weight of the rats showed a significant (*P* < *0.05*) reduction in CP-treated rats compared to both control and AGE-treated animals. Meanwhile, insignificant (*P* > *0.05*) change of the final body weight was recorded in the combined AGE and CP-treated rats compared with CP-treated rats.

### Kidney weights

Both absolute and relative weights of the kidneys of CP-treated rats revealed a significant (*P < 0.001*) difference compared to those of the control rats. However, comparing absolute and relative kidney weights of CP-treated rats, a significant (*P < 0.001*) difference was recorded compared with those of the other groups (Figure 1).

### Biochemical results

**Figure 2:**
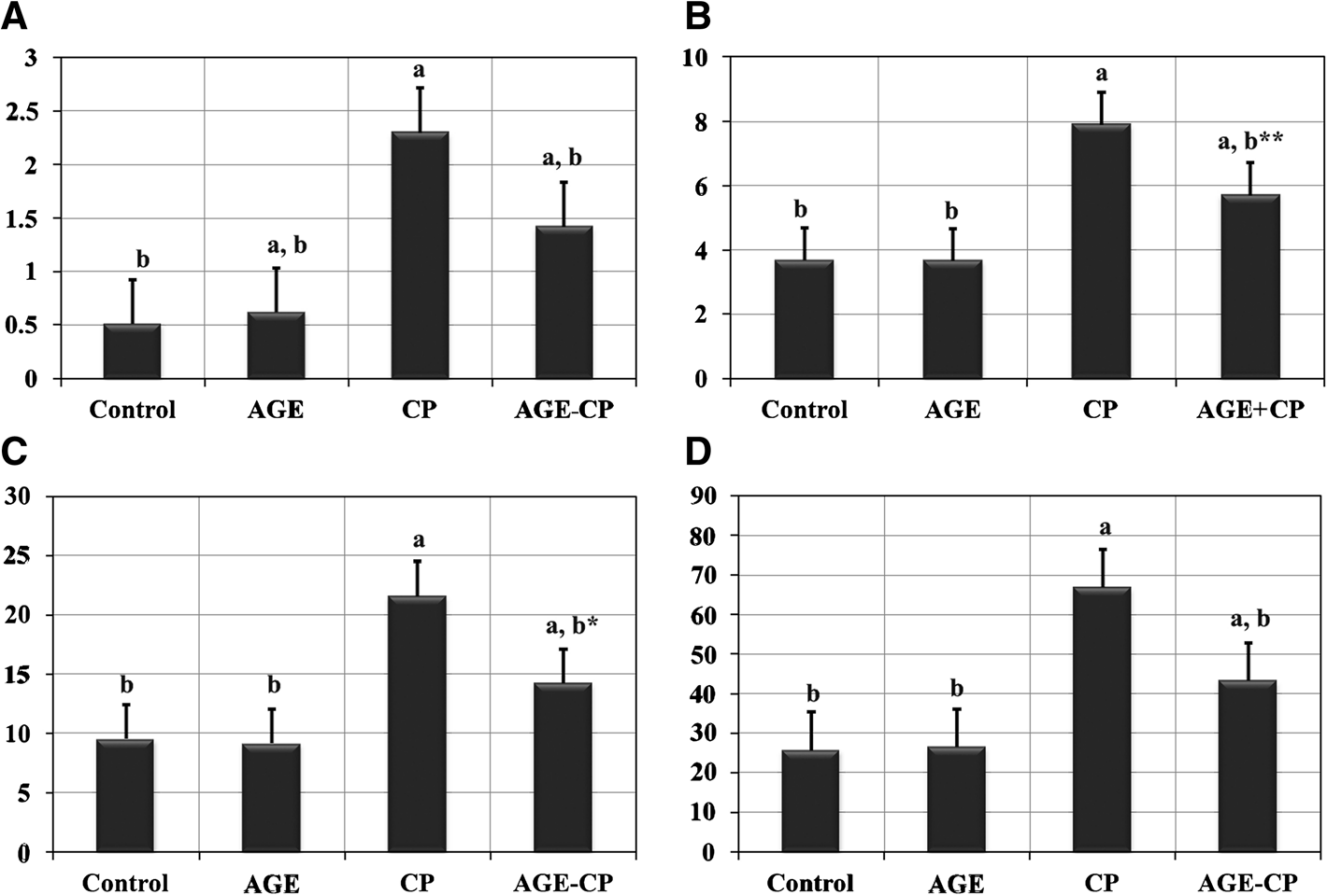
**Effects of cisplatin (CP) and/ or aged garlic extract (AGE) treatment on kidney biomarkers of rats. (A)** shows serum creatinine level. Parameters of serum uric acid are listed in **(B)**. Values of serum BUN are listed in **(C)**. Serum urea levels are shown in **(D)**. Data are expressed as Mean ± SEM (n = 6). a: Significant difference Vs. control group at P.< 0.001. b: Significant difference Vs. cisplatin-treated group at P. < 0.001. BUN: blood urea nitrogen. AGE: aged garlic extract. b*: P. < 0.005; b**: P. < 0.05.

A significant (*P < 0.001*) increase in the serum levels of all kidney biomarkers in CP-treated rats compared to those of control rats. However, the animals pretreated with AGE showed significant (*P < 0.001*) decrease in the serum levels of these biomarkers compared to those of CP-treated rats (Figure 2).

### Lipid peroxidation and antioxidant enzymes

**Figure 3:**
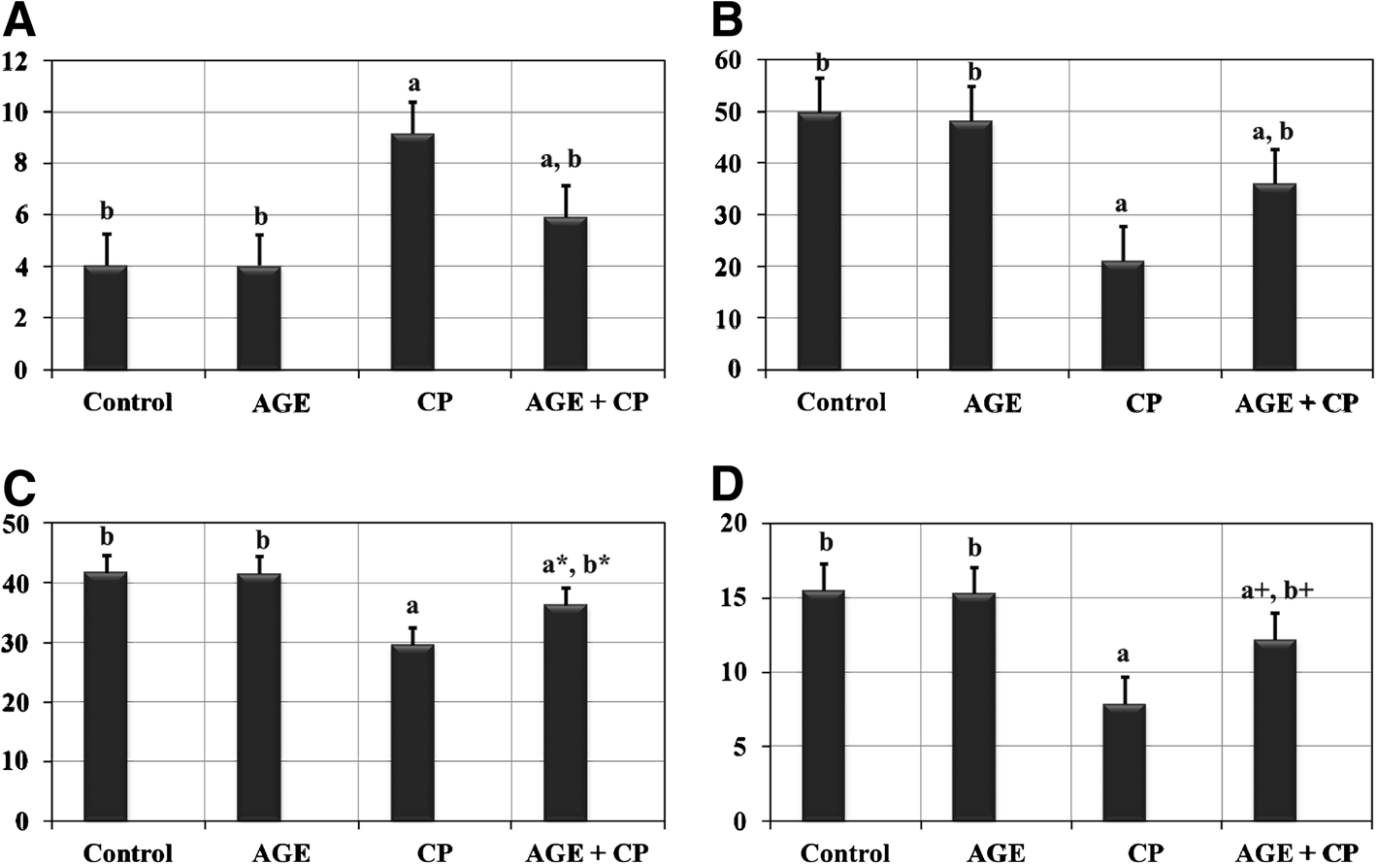
**Effects of aged garlic extract (AGE) administration on malondialdehyde (MDA) level, superoxide dismutase (SOD) activity, catalase (CAT) activity and reduce glutathione (GSH) level in kidneys of Wistar rats treated with cisplatin (CP). (A)** shows tissue level of MDA in different groups. Activities of both SOD **(B)** and CAT **(C)** in renal tissues are listed as well. Tissue level of GSH is listed in **(D)**. Data are expressed as Mean ± SEM (n = 6). a: Significance at *P < 0.001* Vs. control group; b: Significance at *P < 0.001* Vs. cisplatin-treated group. a*: *P = 0.036;* b*: *P = 0.0038*; a+: *P = 0.049*; P+: *P = 0.003*. No significant difference (*P = 0.61 - 0.99*) was recorded between control and AGE (Aged garlic extract)- treated groups in all parameters.

Effects of AGE administration on MDA & GSH levels, SOD & CAT activities in renal tissues of CP-treated rats were listed in (Figure 3). The MDA level in renal tissues of CP-treated rats displayed significant (*P < 0.001*) increase compared with control rats. These changes were attenuated by pre-treatment with AGE. Also, SOD & CAT activities and GSH content revealed significant (*P < 0.001*) reduction in renal tissues of CP-treated rats compared with the control group. Pre-treatment of rats with AGE ameliorated these CP-induced changes in the antioxidant enzymes. A significant (*P < 0.001*) increase in GSH level and SOD activity accompanied with significant (*P < 0.001*) reduction in MDA level were observed in AGE- pretreated renal tissues compared to those of CP-treated group. However, the renal tissue of AGE-treated rats revealed no significant (*P = 0.61-0.99*) differences in levels of MDA & GSH and activity of SOD & CAT compared to control.

### Histopathological results

**Figure 4:**
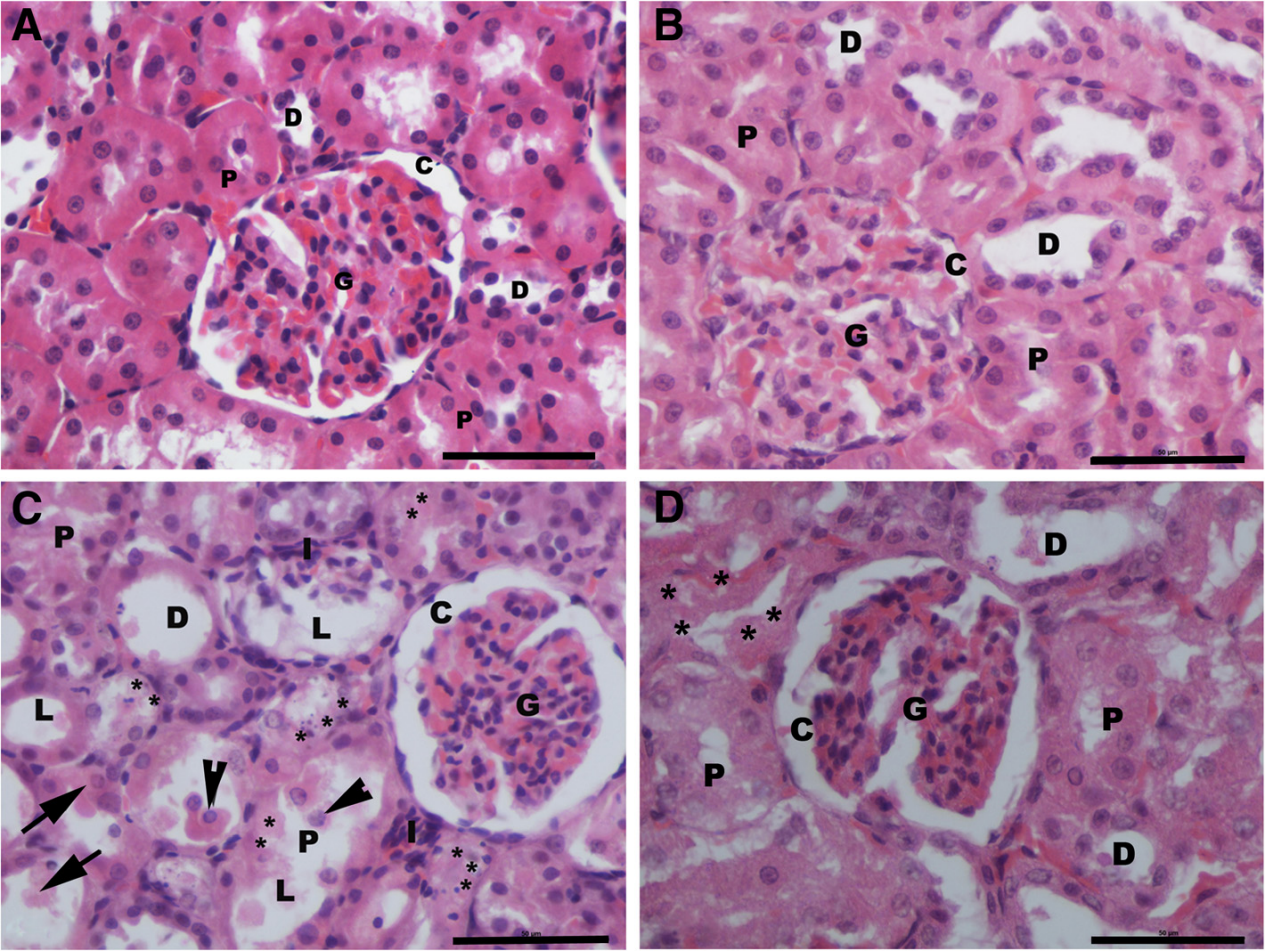
**Light micrographs of the rat’s renal cortex.** Normal cortical structure is observed in the renal sections of both control **(A)** and aged garlic extract-treated **(B)** rats. Glomerular atrophy (G), wide capsular space (C), degenerated (*) proximal tubules (P) with exfoliating cells (arrow head), accumulation of hyaline cast (arrow) within its wide lumina (L), wide lumen of distal convoluted tubules (D) and interstitium inflammatory cell infiltration (I) are noticed in cisplatin-treated rats **(C)**. Wide capsular lumina (C), minimal degenerated (*) proximal tubules (P) and wide distal tubules (D) are seen in the combined aged garlic extract and cisplatin-treated rats **(D)**. H & E stain x600.

Light microscopic examination of renal cortex revealed normal histological structure in control and AGE-treated rats (Figure 4A, B) rats. However, the renal structure of CP-treated rats revealed marked morphological disturbances at the cortico-medullary zone, where wide capsular space, shrinkage hypercellular congested glomeruli, severe tubular degeneration, tubular necrosis, apoptotic nuclear changes, exfoliation of the proximal tubular epithelial cell lining, shedding of the apical microvilli of the proximal tubular cells, luminal dilation with excessive accumulation of homogenous exudate in the proximal tubules, intertubular hemorrhage and inflammatory cell infiltration were observed in the renal cortex. Also, degeneration and luminal dilatation with excessive accumulation of homogenous exudate in distal tubules were seen in most of the distal convoluted tubules as well (Figure 4C). Meanwhile, in AGE pre-treated rats, mild inflammatory cell infiltration and proximal tubular cell necrosis were observed in the renal parenchymal structure (Figure 4D).

**Figure 5:**
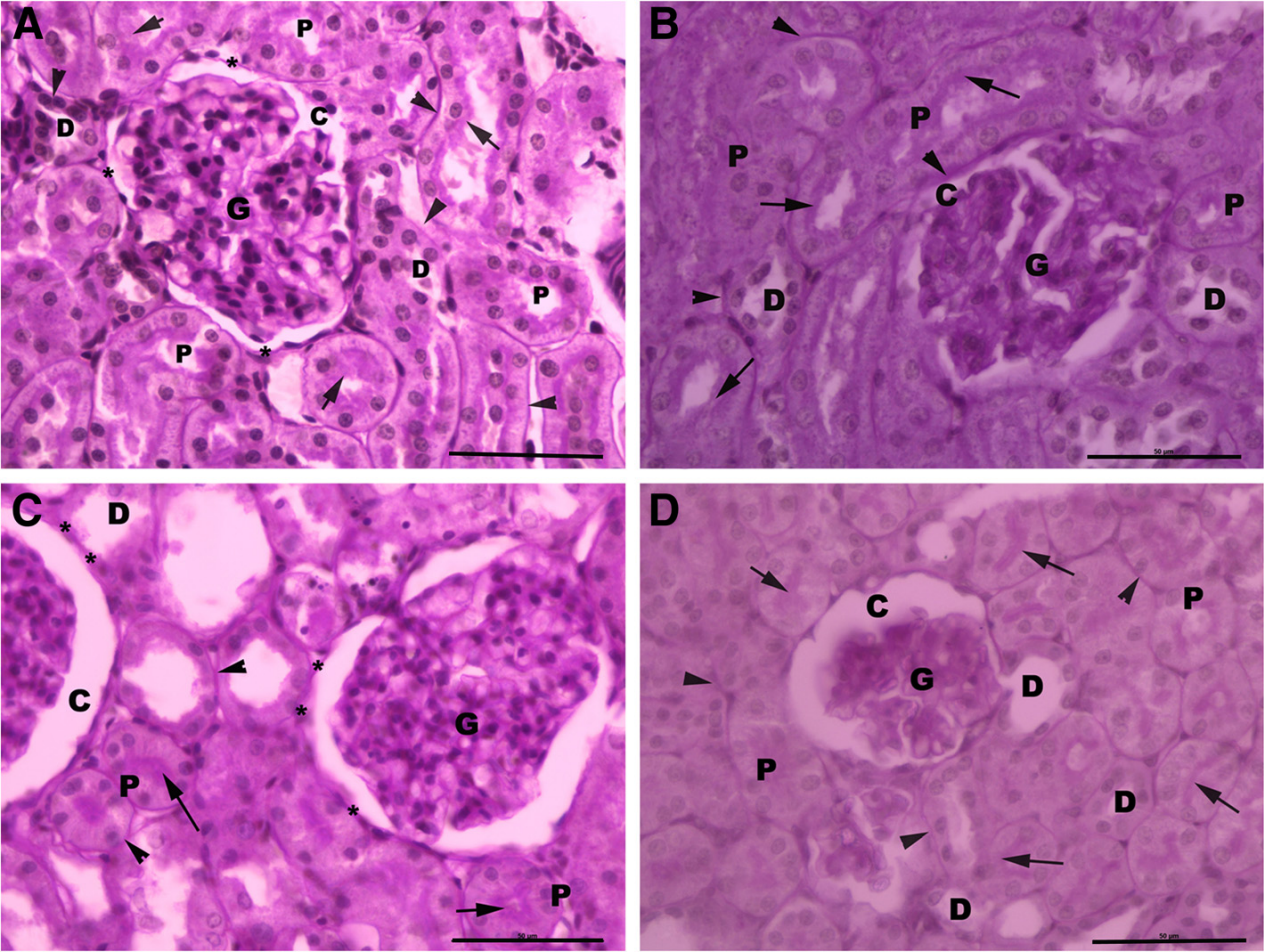
**Light micrographs of the PAS-stained sections of rat's renal cortex.** Strong positive PAS reaction is seen within the brush border (arrow) of proximal tubular epithelial cells (P), basement membrane (arrow head) of both proximal (P) and distal (D) convoluted tubules and in the outer (*) capsular membrane (C) of control **(A)** and aged garlic extract-treated **(B)** rats. Weak reaction is seen in the remaining brush border (arrow), basement tubular membranes (arrow head) and outer capsular membrane (*) in cisplatin-treated rats **(C)**. Strong positive PAS reaction is seen in the brush border (arrow), tubular membrane (arrow head), outer capsular membrane (*) in combined aged garlic extract and cisplatin-treated rats **(D)**. G: glomerulus; C Bowman’s capsule; P: proximal convoluted tubules; D: distal convoluted tubules. PAS stain x 600.

In PAS-stained sections, strong positive PAS reaction was observed in the brush border of proximal convoluted tubules (PCT), basement membrane of both PCT and distal convoluted tubules (DCT), and parietal layer of Bowman’s capsule in control (Figure 5A) and AGE-treated (Figure 5B) rats. While, weak positive PAS reaction was observed in the brush border and basement membranes of the healthy PCT & DCT in CP-treated rats (Figure 5C). A positive PAS reaction was noticed in the brush border and basement membrane of PCT, basement membrane of DCT and outer layer of Bowman’s capsule in renal sections of AGE-pretreated rats (Figure 5D).

### Ultrastructure results

**Figure 6:**
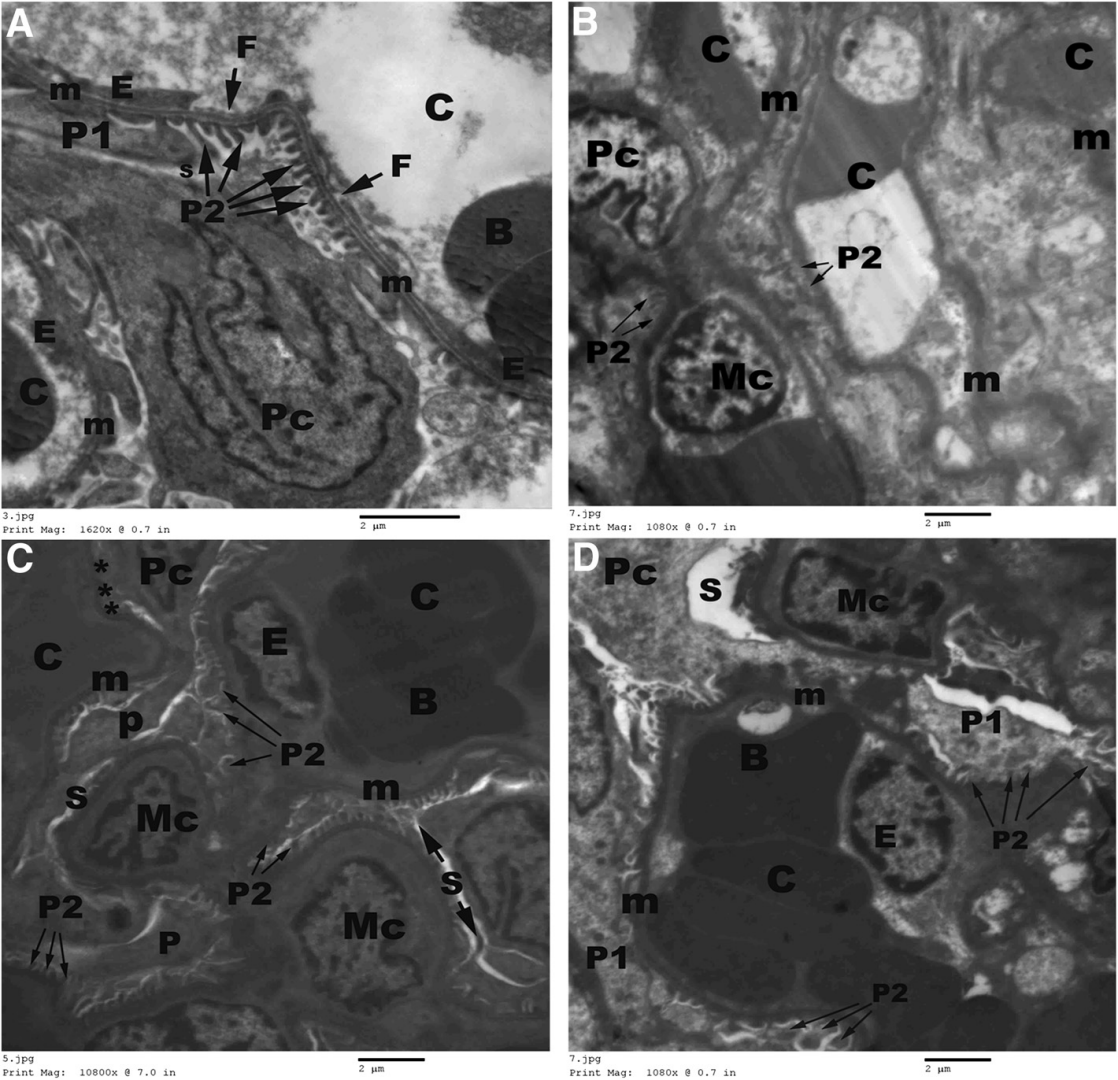
**Electron micrographs of the renal corpuscle of rats.** Normal podocytes (Pc) with primary (P1) and secondary (P2) foot processes, glomerular capillaries (C) with regular continuous basement membrane (m), fenestrated (F) endothelial cell lining (E) and mesangial cells (MC) in-between are seen in renal corpuscle of control **(A)** and aged garlic extract-treated **(B)** rats. Fused foot processes (P2), mesangial hyperplasia (MC) and irregular capillary (C) basement membrane (m) are seen in cisplatin-treated rats **(C)**. Dilated congested glomerular capillaries (C) are seen in combined aged garlic extract and cisplatin-treated rats **(D)**. B: blood cells. BM: tubular basement membrane. S: capsular space. Uranyl acetate-lead citrate Stain.

Electron microscopic examination revealed normal ultrastructure of renal corpuscles in control (Figure 6A) and AGE-treated (Figure 6B) rats, where normal double-layered Bowman’s capsules with narrow capsular space were observed in renal cortex. The outer parietal layer of the Bowman’s capsule exhibited flat cells resting on basement membrane, while its inner visceral layer consisted of large podocytes having eccentric nuclei and many cytoplasmic foot processes resting on the glomerular capillary basement membrane. The glomeruli consisted of many capillary loops and electron-dense mesangial cells in-between. The glomerular capillaries were lined by fenestrated flat endothelial cells and continuous basal lamina of uniform thickness. On the other hand, the renal corpuscles of CP-treated rats showed wide capsular space, fused foot processes, dilated congested capillary loops, irregular capillary basement membrane and mesangial cell hyperplasia with excessive deposited mesangial matrix (Figure 6C). Whereas, in the AGE pre-treated rats, normal podocytes, uniform thickness of the capillary basal lamina and a few dilated congested capillary loops were observed (Figure 6D).

**Figure 7:**
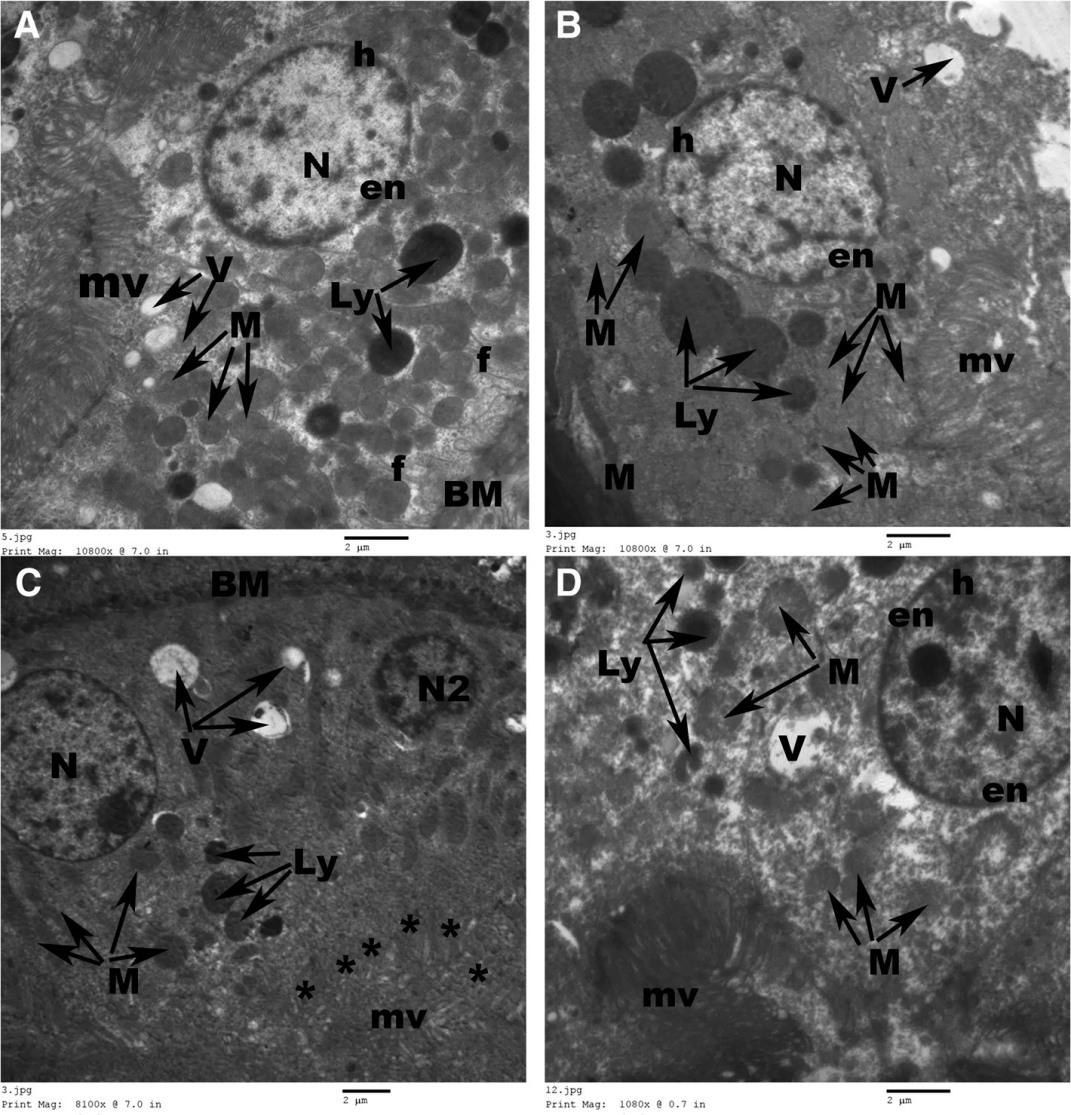
**Electron micrographs of proximal tubular epithelial cells of rats.** Normal cellular ultrastructure is observed in control **(A)** and aged garlic extract-treated **(B)** rats. Marked destruction (*) of apical microvilli (mv), excessive number of lysosomes (Ly), cytoplasmic vesicles (V), irregular basement membrane (BM), degenerated mitochondria (M), marginal nuclear chromatin and apoptotic nuclei (N2) are seen in cisplatin-treated rats **(C)**. Few cytoplasmic vacuoles (V), lysosomes (Ly) are seen in combined aged garlic extract and cisplatin-treated rats **(D)**. N: oval nucleus; n: nucleolus; h: heterochromatin; ne: nuclear envelop. Uranyl acetate-lead citrate Stain.

The epithelial cells lining the PCT of control rat’s kidney revealed normal basal or mid-positioned oval nuclei with regular nuclear envelop and peripheral electron-dense heterochromatin. Their cytoplasm contained numerous rounded mitochondria, many apical pinocytotic vesicles and lysosomes. The PCT cells showed regular basal membrane with many basal infoldings. Their apical surface revealed numerous long apical microvilli projecting within the narrow tubular lumen (Figure 7A). The epithelial lining the PCT of AGE-treated rats revealed an ultrastructure with great similarity to those of the control ones (Figure 7B). However, irregular thick basement membrane with loss of its basal infoldings, rarified cytoplasm containing few pleomorphic mitochondria with electron-dense matrix, many cytoplasmic vacuoles, secondary lysosomes, irregular outlined nuclei with peripherally condensed heterochromatin and destructed apical microvilli were observed in the PCT of CP-treated rats (Figure 7C). On the other side, in the AGE-pretreated rats, normal nuclei, long apical condensed brush border, few lysosomes and a few cytoplasmic vacuoles were seen in the epithelial lining the PCT (Figure 7D).

**Figure 8:**
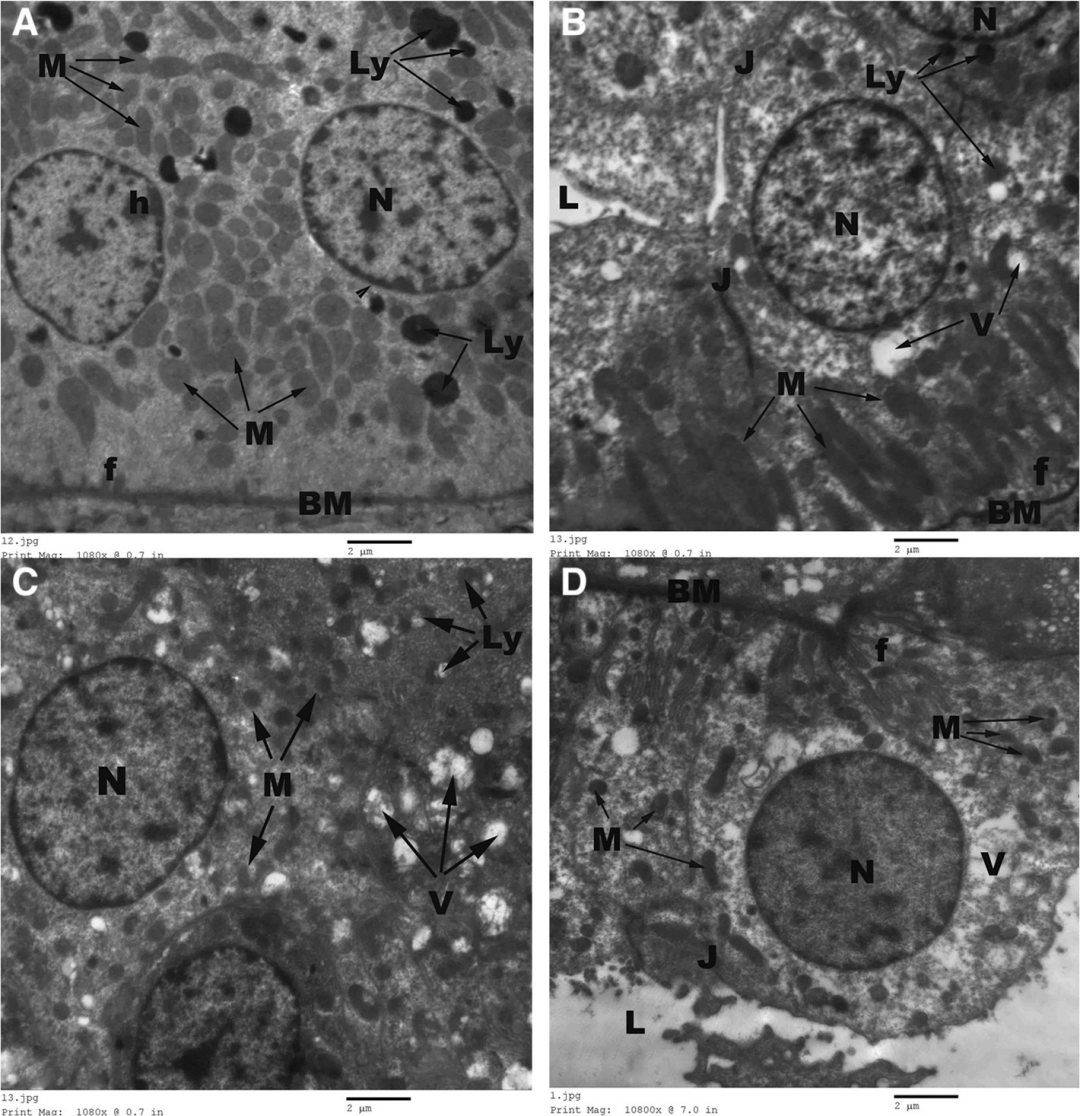
**Normal regular basement membrane (BM), many basal infoldings (f), round regular nuclei (N), numerous elongated basal mitochondria (M), few lysosomes (Ly), apical junctions (J) are seen in control** **(A)** **and aged garlic extract-treated** **(B) rats.** Regular outlined nucleus (N), few small-sized round mitochondria (M), many lysosomes (Ly) and numerous heterogenous cytoplasmic vacuoles (V) are seen in cisplatin-treated rats **(C)**. Regular round nucleus (N), regular basement membrane (BM), numerous basal infoldings (f) and apical tight junctions (J) are seen in combined aged garlic extract and cisplatin-treated rats **(D)**. Uranyl acetate-lead citrate Stain.

The DCT showed normal basic ultrastructure in the renal sections of control rats, where their epithelial cells lining exhibited apical rounded nuclei, thin regular basal lamina with many basal infoldings and many basal elongated mitochondria in-between, few lysosomes and apical tight junctions (Figure 8A). In AGE-treated rats, the epithelial cells lining the DCT revealed apical rounded nuclei, many elongated basal mitochondria, few apical lysosomes, pinocytotic vesicles and apical tight junctions (Figure 8B). However, the DCT of CP-treated rats showed apoptotic nuclear changes with fragmented nuclear envelop, thick basement membrane with few basal infoldings, small-sized degenerated mitochondria, many lysosomes and cytoplasmic vacuoles (Figure 8C). Meanwhile, the DCT of the combined AGE and CP-treated rats revealed regular basement membrane with many basal infoldings, basal oblong mitochondria and apical large euchromatic nuclei with regular nuclear envelop (Figure 8D).

## Discussion

CP exhibits a potent antineoplastic effect against wide variety of solid tumors. However, its uses in the clinical field was markedly limited due to the occurrence of many side effects mainly the nephrotoxicity [1-3]. As the exact mechanisms responsible for CP- induced nephrotoxicity were not fully understood; several studies have been performed to explain how CP could induce toxicity [2,3,11]. To ameliorate the toxic effects of CP, many strategies have been proposed using various natural and synthetic free radical scavengers and antioxidants [3,4,6,7,10,11,21]. Such substances might provide nephroprotection against CP toxicity [11,29].

AGE, a natural and dietary substance, was known to have different active antioxidant organosulfur compounds mainly S-Allylcysteine and allicin [12,13]. These compounds enhanced cellular antioxidant enzymes, increased GSH content in the cells and scavenged the free radicals [12-14]. The protective effect of AGE against doxorubicin-induced cardiotoxicity [16], cadmium-induced toxicity [18] and acrylamide - induced oxidative damage in multiple organs [17] have been previously investigated in literatures. Thus, the present study aimed to evaluate the possible protective effect of AGE against CP-induced oxidative stress and renal damage in adult male rats.

In the present study, single injection of CP (7.5 mg/kg i.p.) revealed a significant reduction in the final body weight of the rats compared to that of control group. In agreement with the results of this study, a significant reduction in the body weight of CP-treated rats was observed 3–5 days after single i.p injection (5–7.5 mg/kg) of CP [10,21,30]. The reduction of the body weight in CP-treated rats might be in part due to the direct toxic effect of CP on renal tubules that caused reduction in water reabsorbtion and excessive sodium excretion with subsequent polyuria, dehydration and reduction of body weight [7,20,29]; or might be due to gastrointestinal toxicity with subsequent decrease in appetite, ingestion and assimilation of food [7,8]. On the other hand, an obvious gain in the body weight was observed in AGE pre-treated rats. Such effect gave an approval on the possible protective effect of AGE against the toxicity of CP on renal and intestinal tissues.

In this study, a remarkable decrease in the absolute weight and a significant increase in the relative weight of the kidneys were observed in CP-treated rats. These results were in accordance with that of Mansour et al. [6]; Arhoghro et al. [8]; Anusuya et al. [15]; Nasr [21]; Ali and Al-Moundhri [29] who stated that a single CP injection (7.5 mg/kg i.p.) resulted in loss in the absolute and increase in relative weights of the kidneys in rats. The reduction in absolute kidney weight in CP-treated rats could be due to the damage in renal tissues and reduction in their functions [31]. However, the increase in reno-somatic index (kidney weight/body weight ratio) might be due to the edema of renal parenchyma since CP was known to cause renal inflammation [6,9,15]. The oral intake of AGE returned the relative kidney weight of CP-treated close to those of control rats. This protective effect of AGE on the kidney weights could be attributed to its anti-inflammatory effect that decreased the renal edema induced by CP [15]. However, the AGE showed no effect on the kidney weight of normal rats. This indicated that, the AGE had no influence on the morphology and functions of the normal kidney.

Serum creatinine, urea, uric acid and BUN levels were considered the main parameters that determine the glomerular filtration rate [20]. In the present study, nephrotoxicity of CP was evident from the elevated levels of serum urea, uric acid, creatinine and BUN levels. The elevation in the serum levels of these renal biomarkers might be due to the impaired renal functions [15], tubular obstruction, and/or the back-leakage of the renal tubules [20]. Such functional disturbance in CP exposed rats could indicate the ability of CP to inhibit protein synthesis in the tubular cells [9] or to initiate lipid peroxidation and generate free radicals in renal tubules [3,4,6,7]. Similar findings were previously reported in different studies [5,6,8,20]. The elevation in the kidney biomarkers was due to the direct toxic effect of CP on the glomerular and tubular structures through the generation of ROS. Contraction of the mesangial cells with subsequent alteration in the filtration surface area resulted from the generation of ROS [32].

However, the serum BUN, urea, uric acid and creatinine levels revealed marked improvement in the combined AGE + CP- treated rats. These results confirmed the ability of AGE to improve or normalize the renal function through its organosulfur compounds that could increase the antioxidant effect and decrease lipid peroxide levels by scavenging the free radicals and increasing intracellular concentration of glutathione [15]. In agree with the results of this study, feeding of AGE provided a protective effect in other experimental studies [5,15-18].

In our study, pre-treatment with AGE produced significant reduction in MDA level and significant elevation of SOD, CAT activities and GSH levels of the renal tissues in CP-treated rats. These findings provide an evidence about the antioxidant effect of AGE against CP-induced oxidative stress and lipid peroxidation. In agree with the results of this study, reduction in the activity of antioxidant enzymes (CAT & SOD), increased lipid peroxidation (MDA) and depletion of GSH in renal tissues were implicated in the pathogenesis of CP nephrotoxicity [4-6].

ROS produced by xanthine-xanthine oxidase system, mitochondria and NADPH oxidase in cells directly act on lipid, proteins and DNA of the cell and destroy them [7]. However, animals pre-treated with AGE showed significant improvement in biochemical. histological and ultrastructural changes in renal tissues induced by CP. Many studies have demonstrated that AGE prevents the oxidative stress and exerts a protective effect against different toxic agents through its powerful antioxidant and free radical scavengers [14-17].

In our study, the histopathological changes confirmed the biochemical findings, where reduction in the size of the glomerular capillary tufts, dilatation of Bowman’s capsule, degeneration, necrosis and detachment of the proximal tubular epithelial cell lining, shedding of the apical microvilli and accumulation of homogenous exudates within the dilated tubular lumina of both proximal and distal convoluted tubules were observed in renal sections at the corticomedullary zone of CP-treated rats. Some of the tubules were totally degenerated and cellular details were completely lost. Distal tubular cells were also affected. Consistent with the results of the present study, Mansour et al. [6]; Abdelmaguid et al. [27], Nasr [18]; Ali and Al-Moundhri, [28]; Chirino et al. [29] reported similar findings in rats exposed to different doses of CP.

Glomerular atrophy, degeneration of the tubular epithelium and interstitial inflammatory cell infiltration were the main histopathological findings of CP-treated renal tissues. Although glomerular pathology was not identified in the majority of CP-treated animals [7]; other studies have reported glomerular damage in CP-induced renal injury [10,33,34]. In our study, administration of CP caused not only an extensive degeneration of the tubular epithelium but also glomerular damage as well. The histopathological observations of the present study agreed with those of the previous investigations [18,26,27,33,34].

In addition to the histopathological findings, prominent ultrastructural changes were observed in the proximal tubular epithelial cells, where marked tubular degeneration with distinct apoptotic changes, loss of apical microvilli, irregularity of basement membrane, rarification of cytoplasm, marginal condensation of nuclear chromatin, vesiculation in the mitochondria and presence of many lysosomes were observed in CP-treated renal tissues. Irregular glomerular basement membrane, fusion of foot processes, increase mesangial matrix with proliferation of mesangial cells were observed in the electron microscopic examination of CP-treated rats as well. The ultrastructure changes in our work were in accordance with the observations of the prior investigations [10,11,35]**,** where cytoplasmic vacuolization, dissociation of mitochondria from basal infoldings, loss of basal infoldings, many pinocytotic vesicles and lysosomes in apical region of the proximal tubular epithelial cells were reported in renal sections of CP-treated rats.

Both histopathological and ultrastructural findings of the present study revealed the direct toxic effect of CP on the renal tissues. The histopathological changes obtained in the present study run parallel with the reports documented by Abdelmaguid et al. [10], Nasr [21]; Chirino et al. [30]; Ali and AL-Moundhri [29] who found tubular necrosis and glomerular atrophy in CP-treated animals. Reduction or complete absence of the apical microvilli induced an impairment in amino acids and glucose absorption. In addition, presence of many mitochondria of variable length with dense matrix along basolateral folding was indicative of respiratory impairment. Such mitochondrial changes might be due to the response of mitochondria to overcome the direct stress of CP on renal tissues. The author added that, condensation of nuclear chromatin, presence of necrotic debris in the tubular lumen with tubular damage and appearance of lysosomal bodies in any cell might be indicating the degenerative activity and the onset of necrosis [35].

However, in combined AGE + CP-treated animals, normal foot processes, glomerular mesangium, preserved apical microvilli, restored nuclear structure with no evidence of inflammatory cell infiltration were noticed in the present study.

Inflammation was considered as an important mechanism in CP- induced nephrotoxicity in which TNF-α showed a central role [2]. Thus, absence of infiltration of the inflammatory cells in combined AGE + CP-treated renal tissues, in contrast to CP-treated tissues, could be considered as a prove to the anti-inflammatory effect of AGE [34]. To our knowledge, this study was the first one demonstrating the protective effect of AGE on CP-induced nephrotoxicity at biochemical, histomorphological and ultrastructural levels.

Apoptosis is an important way of cell death in normal and pathological processes. In the present study, different apoptotic changes, in the form of membrane budding, cell shrinkage and chromatin condensation, were observed in CP-treated renal tissue. The process of apoptosis was explained by three different pathways: extrinsic pathway, mitochondrial/intrinsic pathway [2]. The authors added that, the mitochondrial/intrinsic pathway revealed an important role in CP-induced nephrotoxicity. Thus, the protective role of AGE on CP-induced nephrotoxicity might be primarily due to its anti-apoptotic property.

The findings of present study demonstrated that most of biochemical, histological and ultrastructural alterations induced by CP treatment could be ameliorated by the pre-treatment with AGE, where the morphological and biochemical changes in AGE + CP-treated rats were markedly less than those observed in rats treated with CP alone.

## Conclusion

From the findings of the present study, we can conclude that, AGE has an ameliorative effect against CP-induced oxidative stress and renal damage through its antioxidant, anti-inflammatory and antiapoptotic properties. However, the protective Effect of AGE against both antineoplastic activity of CP and the effect of CP on the mitochondrial intrinsic pathway did not investigate in this work. Thus, these points need further studies to be fulfilled.
